# Virus-like particles containing a prefusion-stabilized F protein induce a balanced immune response and confer protection against respiratory syncytial virus infection in mice

**DOI:** 10.3389/fimmu.2022.1054005

**Published:** 2022-12-12

**Authors:** Jin Luo, Huan Qin, Lei Lei, Wange Lou, Ruitong Li, Zishu Pan

**Affiliations:** State Key Laboratory of Virology, College of Life Sciences, Wuhan University, Wuhan, China

**Keywords:** respiratory syncytial virus, virus-like particles, vaccine, prefusion F protein, postfusion F protein, baculovirus insect cell expression system

## Abstract

Respiratory syncytial virus (RSV) is a serious respiratory pathogen in infants and young children worldwide. Currently, no licensed RSV vaccines are available. In this study, we explored stable prefusion conformation virus-like particles (Pre-F VLPs) as RSV vaccine candidates. RSV fusion (F) protein mutants were constructed to form stabilized Pre-F or postfusion (Post-F) configurations. VLPs containing Pre-F or Post-F protein were generated using a recombinant baculovirus (rBV)-insect cell expression system. The assembly and immunological properties of Pre-F or Post-F VLPs were investigated. Pre-F and Post-F VLPs contained antigenic sites Ø and I of pre- and postfusion conformations, respectively. Compared with Post-F VLPs, immunization with Pre-F VLPs elicited upregulation of IFN-γ, IL-2 and IL-10 and downregulation of IL-4 and IL-5 cytokine production in mice. A high percentage of CD25^+^ Foxp3^+^ cells or a low percentage of IL-17A-producing cells among CD4^+^ T cells was observed in the lungs of mice vaccinated with Pre-F VLPs. Importantly, immunization with Pre-F VLPs induced a high level of RSV neutralizing antibody and a balanced immune response, which protected mice against RSV infection without evidence of immunopathology. Our results suggested that Pre-F VLPs generated from rBV-insect cells represent promising RSV vaccine candidates.

## Introduction

Human respiratory syncytial virus (RSV) was ascertained as a leading cause of bronchiolitis in infants as early as 1956 (1, 2). RSV infection causes a substantial disease burden in infant, immunocompromised, and elderly populations (3–5). Natural RSV infection does not induce sustained immunity, and repeated infections occur throughout life (6, 7). Therefore, it is particularly urgent to develop effective treatments and vaccines for RSV infection. Despite extensive efforts, no licensed RSV vaccines are available. Vaccination with formalin-inactivated RSV (FI-RSV) in the 1960s led to vaccine-enhanced disease (VED) upon RSV challenge (8–10), thus impeding RSV vaccine development. Intensive investigation showed that VED exhibited a strong relationship with the exaggerated Th2-type immune response and the poorly neutralizing antibodies upon RSV infection (11–14). Therefore, induction of a Th1-biased, balanced immune response and high neutralizing antibody production are critical for an effective RSV vaccine (15).

The RSV fusion (F) and attachment (G) glycoproteins presented on the virions are the major targets for RSV vaccine candidates (16–19). The F glycoprotein, which induces high neutralizing antibody titres and specific cellular immunity, provides immune protection and cross-protection against different RSV strains (20, 21). Crystal structures of both prefusion (Pre-F) and postfusion (Post-F) forms provided structural insights into the antigenicity of RSV F protein (22, 23), demonstrating that vaccines based on the Pre-F configuration represent promising next-generation vaccine candidates (24–26). A Pre-F form of the F protein contains an antigenic site Ø, which is not present in its Post-F conformation (27). Specific monoclonal antibodies directed to site Ø exhibited good RSV neutralizing ability (22). The engineered Pre-F protein exhibited enhanced physical and antigenic stability relative to DS-Cav1 (28, 29). A stabilized Pre-F protein elicited significantly increased neutralizing antibody titres compared with the Post-F form in animals, suggesting that a stable Pre-F protein represents a promising strategy for RSV vaccine candidate (27, 30–32).

Virus-like particles (VLPs) are effective, safe and promising vaccine platforms (33, 34). VLPs are genetically engineered complexes of multiple copies of protein antigens in a virus-like structure; VLPs lack viral genetic material and therefore cannot replicate (35, 36). Commercial VLP-based licensed vaccines are available against human papilloma and hepatitis B viruses (36). RSV glycoproteins presented as VLPs are highly immunogenic and confer protection against RSV infection (37–40). In the present study, we produced and characterized VLPs containing the stable prefusion and postfusion forms of the RSV F protein using an rBV-insect cell expression system. Immune responses and protection against RSV challenge induced by these VLPs were investigated in BALB/c mice.

## Materials and methods

### Cells, viruses, and preparation of ultraviolet (UV)-inactivated virus and antibodies

HEp-2 and Vero cells were obtained from the China Center for Type Culture Collection (CCTCC; Wuhan, China) and grown in Dulbecco’s modified Eagle’s medium (DMEM) supplemented with 10% foetal bovine serum (FBS, Gibco), 100 IU of penicillin, and 100 mg/ml streptomycin at 37°C and 5% CO_2_. The respiratory syncytial virus (RSV) A2 strain was maintained in our laboratory. *Spodoptera frugiperda* 9 (Sf9) cells were obtained from the American Type Culture Collection (ATCC, Rockville, MD, USA) and cultured at 27°C in SF-900 II serum-free medium (SFM) (Invitrogen, USA), 100 IU penicillin and 100 mg/ml streptomycin. RSV was propagated in HEp-2 cells, and virus titres were quantified in Vero cells. RSV purification and inactivation by UV light was performed as previously described (18, 39). Briefly, RSV-infected HEp-2 cells were sonicated, clarified by centrifugation (1,200 × g for 30 min at 4°C) and concentrated by ultracentrifugation (120,000 ×g at 4°C for 6 h). The resultant precipitate was resuspended in phosphate-buffered saline (PBS) for RSV titration. For RSV purification, the resultant precipitate was resuspended in 10% sucrose in PBS; layered on top of a discontinuous sucrose gradient composed of 2 ml of 60%, 45%, and 30% sucrose (in PBS); and then centrifuged at 160,000 × g in a SW28 rotor for 2 h. The visible virus band between the 30% and 45% sucrose layers was collected for subsequent assays. For virus inactivation, 0.5 ml of a purified RSV suspension (10^6^ PFU/ml) in a 35-mm petri dish was irradiated with ultraviolet (UV) light for 40 min, and the efficacy of UV inactivation was examined by determining the infectivity of inactivated RSV in Vero cells using a plaque assay. Mouse anti-F monoclonal antibody (mAb) clone 131-2A (Millipore, Temecula, USA), human anti-F mAb clone D25 (Cambridge Biologics, Boston, USA), goat anti-mouse IgG coupled to horseradish peroxidase (HRP) (Abclonal, Wuhan, China), and goat anti-human IgG coupled to HRP (Abclonal) were used in VLP binding assays.

### Construction of plasmids and recombinant baculoviruses

**Figure 1:**
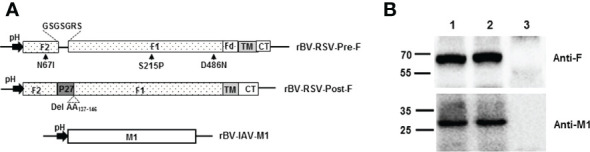
Construction of recombinant baculoviruses and expression of proteins in Sf9. **(A)** Schematic representation of the recombinant baculovirus vectors used in the present study. pH, polyhedrin promoter of baculovirus; **(B)** Coexpression of the RSV F and H1N1 M1 proteins in Sf9 cells. Sf9 cells were infected with the indicated rBVs, harvested at 3 days postinfection (p.i.) and subjected to Western blot analysis using a mouse monoclonal anti-RSV F antibody and a mouse polyclonal anti-H1N1 M1 antibody. M, Molecular marker; Lane 1, rBV-RSV-Pre-F/M1; Lane 2, rBV-RSV-Post-F/M1; Lane 3, Sf9 cells.

The Pre-F and Post-F forms of RSV F protein (GenBank: ACO83301.1) were prepared as previously described (29, 41, 42). To obtain the stable prefusion F conformation protein, the site mutations N67I, S215P, and D486N were introduced into the F fragment. Then, the sequence encoding the T4 fibritin trimerization domain (foldon, SAIGGYIPEAPRDGQAYVRKDGEWVLLSTFL) was inserted into the position at amino acids 513 to 514 of the F sequence. Subsequently, the P27 (amino acids 110-136) of the F protein was substituted with the linker (GSGSGRS) to generate a prefusion-stabilized F construct with the transmembrane (TM) and cytoplasmic domains (CT) in the F_514-574_ location (**Figure 1A**). Similarly, the stable postfusion F form was constructed by deleting the sequence encoding amino acids 137 to 146 of the F protein. The sequence encoding the Pre-F, Post-F conformation, or IAV M1 gene (GenBank: ACP44152.1) was codon optimized for insect codon usage and synthesized by Sangon Biotech (Shanghai, China).

The Pre-F fragment was PCR-amplified using primers Pre-F/F and Pre-F/R with the *EcoR* I and *Xbal* I enzyme sites. The amplified product was digested with *EcoR* I and *Xbal* I and cloned into the *EcoR* I/*Xbal* I-digested pFBDM vector under the control of the promoter polyhedrin (pH) to generate the plasmid pFBDM-Pre-F. Similarly, the Post-F fragment or IAV M1 gene was amplified using primers Post-F-F/R or M1-F/R with the *EcoR* I and *Xbal* I enzyme sites and cloned into the pFBDM to generate the plasmid pFBDM-Post-F or pFBDM-M1, respectively. All specific primers used are listed in **Table 1**.

**Table 1:** Oligonucleotides in specific primers for the construction of recombinant plasmids.

Primer	Nucleotide sequence (5′ - 3′)	Enzyme
**PreF-F**	CGGAATTC **GCCACC**ATGGAACTGCTGA	*EcoR I*
**PreF-R**	GCTCTAGATTAGTTTGAGAAAGCGATGTTGTTGATTCC	*Xbal I*
**PostF-F**	CGGAATTC **GCCACC**ATGGAGTTGCTA	*EcoR I*
**PostF-R**	GCTCTAGATTAGTTACTAAATGCAATATTATTTATACCAC	*Xbal I*
**M1-F**	CGGAATTC **GCCACC**ATGAGCCTGCTGACCGAGGTGGAGACCTAC	*EcoR I*
**M1-R**	GCTCTAGATCACTTGAAACGCTGCATCTGCAC	*Xbal I*
**RSV N-F**	GGTGGAGAAGCAGGATTCTACCATATATTG	*For qRT−PCR*
**RSV N-R**	CTGTATTCTCCCATTATGCCTAGGCC	

Underlined sequences represent restriction enzyme sites. Bolded regions indicate Kozak coding sequences.

The identities of the plasmid constructs were verified by sequencing and subsequently used to generate recombinant baculovirus rBV-RSV-Pre-F, rBV-RSV-Post-F, or rBV-IAV-M1, as previously described (17, 43). In brief, the plasmids pFBDM-Pre-F, pFBDM-Post-F, and pFBDM-M1 were transformed into competent *E. coli* DH10 MultiBac cells to generate recombinant bacmids. The resultant bacmid DNA was separately transfected into Sf9 insect cells to obtain the corresponding recombinant baculovirus designated rBV-RSV-Pre-F, rBV-RSV-Post-F, or rBV-IAV-M1.

### Production and purification of chimeric VLPs

The RSV Pre-F VLPs and Post-F VLPs were produced by Sf9 cells coinfected with rBV-RSV-Pre-F and rBV-IAV-M1 or rBV-RSV-Post-F and rBV-IAV-M1 (MOI=1 for each rBV). rBV-infected Sf9 cells were cultured in SF-900 II SFM at 27°C for 3 days. Then, the cultured Sf9 cells were collected by centrifugation and lysed and frozen once at -80°C, and the cell lysates were clarified at 5000 rpm for 30 min. The VLP-containing supernatant was centrifuged at 60000×g and 4°C for 4 h, and the pellets were collected and resuspended in phosphate buffer (0.15 M NaCl, 0.05 M phosphate, pH 7.2). The protein samples were purified using HiPrep Sephacryl S-500 HR (GE Healthcare, Freiburg, Germany) in phosphate buffer at a flow rate of 0.5 ml/min as recommended by the manufacturer. Purified VLPs were quantified using the Bradford protein assay kit (Sangon Co., Ltd.) according to the manufacturer’s instructions.

### SDS−PAGE, Western blot analysis and electron microscopy observation

The expressed proteins were characterized by SDS−PAGE, western blotting and electron microscope observation as previously described (44). In brief, the prepared samples were separated on 12% polyacrylamide gels and stained with Coomassie blue R250 or transferred onto PVDF membranes for western blot analysis using a mouse anti-F mAb clone 131-2A (Millipore) or a mouse anti-M1 mAb clone 36H4 (Immune Tech, New York, USA). The purified RSV Pre-F or Post-F VLPs adsorbed onto copper grids were negatively stained with 2% phosphotungstic acid and observed with a transmission electron microscope (JEM-2100, JEOL, Tokyo, Japan).

### Immunization and challenge of mice

Specific-pathogen-free (SPF) female BALB/c mice (Wuhan University Center for Animal Experiments) aged 6-8 weeks old were intramuscularly (i.m.) immunized thrice at 2-week intervals with 10 μg VLPs in 100 μl (45, 46). For the UV-RSV control, mice were immunized i.m. with 1×10^5^ PFU of purified UV-RSV in 100 μl (18). For the PBS control, mice were inoculated i.m. with 100 μl PBS. Blood was collected by tail vein puncture during preimmunization and at 2 weeks after the final immunization for antibody detection, and splenocytes were isolated for cytokine detection.

For histological analysis, mice were intranasally (i.n.) challenged with 3×10^6^ PFU of RSV A2 in 100 μl at 2 weeks after the final immunization. The whole lungs of three mice were harvested on Day 4 following RSV challenge, immersed in 4% paraformaldehyde, embedded in paraffin and sectioned. The tissue sections were stained with haematoxylin and eosin (H&E) for routine evaluation and with periodic acid-Schiff (PAS) staining of amylase-treated tissue for observation of mucus secretion. The lung inflammation scores were defined as previously described (16, 44), where 0 indicates no inflammation, 1 indicates minimal inflammation, 2 indicates mild inflammation, 3 indicates moderate inflammation, and 4 indicates marked inflammation. Mucus hypersecretion scores in airways were defined as follows: 1-no mucus detectable, 2-rare mucus, 3-moderate mucus accumulation, 4-severe mucus production (47).

### ELISA

Virus-specific IgG, IgG2a, and IgG1 antibodies in mouse sera were determined by enzyme-linked immunosorbent assay (ELISA) using RSV as the coating antigen (18, 48). Briefly, each well of a 96-well plate was coated with 100 μl of purified inactivated RSV (1×10^5^ PFU/well). Serial dilutions of mouse sera in PBS/Tween-20 (PBS-T) containing 1% BSA were added to the wells and incubated at 37°C for 1 h. An HRP-conjugated goat anti-mouse IgG, IgG2a, or IgG1 mAb (Abclonal) was used as the secondary antibody.

Cytokine concentrations in the splenocytes or lung homogenates were quantified by ELISA as previously described (17). Regarding splenocyte cytokines, splenocyte suspensions were prepared from the spleens of experimental mice using Mouse 1×Lymphocyte Separation Medium (Dakewe Biotech, Beijing, China) according to the manufacturer’s protocol. Splenocytes (1×10^6^) were cultured in a 24-well culture plate (Corning, NY, USA) in the presence or absence of 10^5^ PFU of purified UV-RSV. The culture plate was maintained in a 5% CO_2_ incubator at 37°C for 72 h, and the supernatants were then collected and stored at -80°C for subsequent assays. For lung cytokine detection, lung tissues were collected on Day 4 postchallenge (p.c.) and homogenized. After centrifugation, the supernatants were collected and stored at -80°C for the subsequent assay. Th1 (IFN-γ, IL-2), Th2 (IL-4, IL-5), IL-10 and IL-17A cytokines present in the supernatants were quantitatively measured using commercially available ELISA kits (Bio Legend, Camarillo, CA, USA).

Antibody binding to purified VLPs was performed as previously described (49). Briefly, equivalent amounts of Pre-F or Post-F VLPs were added directly to the microtiter wells (1 μg of total VLP protein in 100 μl) and incubated at 37°C for 16 h. After washing thrice with PBS, different concentrations of selected antibody (anti-F131-2A or anti-F D25) were added to each well and then incubated for 2 h at room temperature (RT). After three washes in PBS, the secondary antibody (goat anti-mouse IgG-HRP or goat anti-human IgG-HRP) diluted with PBS containing 1% BSA was added to each well (100 μl/well) and then incubated at RT for 1.5 h. TMB (3,3’,5,5’-tetramethylbenzidine; Sigma) substrate in a 100 μl volume was added to each well. After 15 to 20 min of incubation at RT, the reaction was stopped by adding 100 μl of 2 M H_2_SO_4_ to each well, and the optical density at 450 nm was measured using an ELISA reader (Multidkan MK3; Thermo Fisher Scientific).

### RSV immunoplaque and neutralization antibody assays

RSV neutralizing antibody titres were determined by a plaque reduction assay as previously described (18, 39). Briefly, mouse sera were inactivated at 56°C for 30 min and then serially diluted 2-fold in DMEM. Purified RSV was diluted to approximately 100 PFU in 100 μl and added to the diluted sera in 100-μl aliquots. The virus-serum mixture or a virus-DMEM control was incubated at 37°C for 1 h. Then, the mixture was added to prewashed Vero cells in 24-well plates. After 2 h of incubation, the mixture was removed, and 1 ml of methylcellulose overlay (1 volume of 2 ×DMEM containing 4% FBS, 2% penicillin streptomycin, and 1 volume of 2% methylcellulose) was added to each well. The plates were incubated at 37°C for 3 to 5 days, and the plaques were stained as previously described (39). The neutralization titre was defined as the log_2_ of the reciprocal of the highest dilution of serum that reduced the virus titre by 50%.

### Quantitative real-time (qRT)-PCR

RSV load in the lung was quantified by qRT−PCR (18). Total RNA of lung tissues was extracted using RNA Pure reagent (Aidlab, Beijing, China) and reverse transcribed into cDNA using a reverse transcription kit (Toyoba, Osaka, Japan) according to the manufacturer’s instructions. RSV N gene copies were quantified using 2×SYBR green master mix (Novoprotein, Shanghai, China) in a 7500 Real-Time PCR System (Applied Biosystems, USA). The qRT−PCR primer sequences are listed in **Table 1**.

### Flow cytometry

Cytokine staining was performed as previously described (13, 18, 50). Lung cells were surface-stained with mAbs specific to CD4-FITC (Clone RM4-5) and CD25-APC (Clone PC61) (BioLegend, San Diego, CA). After fixation and permeabilization, the cells were intracellularly stained with PE-labelled anti-mouse Foxp3 (Clone MF-14) or IL17A mAb (Clone TC11-18H10.1) (BioLegend). Then, the stained cells were analyzed by flow cytometry (Beckman Coulter CytoFlex, USA). Data were analysed using CytoFlex software and are presented as the percentage of CD25^+^ Foxp3^+^ cells or IL17A-producing cells among CD4^+^ T cells. All gating strategies are specified in the **Supplementary Figure S1**.

### Statistical analysis

Comparisons of various groups were accomplished by Student’s *t* tests or analysis of variance (ANOVA) followed by the Tukey test or nonparametric Kruskal−Wallis test. A *P* value of less than 0.05 was considered statistically significant.

## Results

### Preparation and characterization of chimeric VLPs

**Figure 2:**
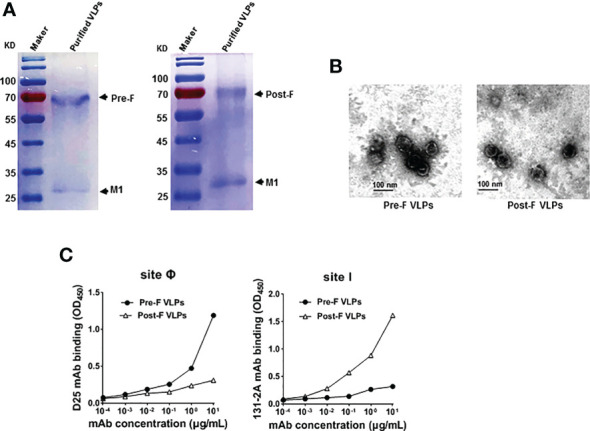
Purification and characterization of Pre-F VLPs and Post-F VLPs. **(A)** Purified proteins were subjected to 12% SDS−PAGE and stained with Coomassie blue. M, Molecular marker; Lane 1, Pre-F VLPs; Lane 2, Post-F VLPs. **(B)** Electron microscopic analysis of Pre-F VLPs and Post-F VLPs. Scale bar, 100 nm. **(C)** Monoclonal antibody specifically binding to purified VLPs. Pre-F and Post-F VLPs containing equivalent amounts of F protein, as determined using mAb D25 or mAb 131-2A as the primary antibody and goat anti-human IgG-HRP for D25 (left) or goat anti-mouse IgG-HRP for 131-2A (right) as the secondary antibody, respectively. Antibodies and sites are indicated in the figure. OD, optical density.

Recombinant baculovirus was constructed as described in the Materials and Methods section (**Figure 1A**). Pre-F or Post-F VLPs were produced by Sf9 cells infected simultaneously with rBV-RSV-Pre-F and rBV-IAV-M1 or rBV-RSV-Post-F and rBV-IAV-M1, respectively. After 72 h of culture, the resultant supernatants of infected Sf9 cells were harvested for analysis of F and M1 expression by western blotting. The data showed that the engineered Pre-F, Post-F or IAV M1 protein could be expressed efficiently from infected Sf9 cells and that the expected molecular weights of RSV F (~63 kDa) and IAV M1 (~28 kDa) proteins were successfully detected (**Figure 1B**). SDS−PAGE analysis showed that the prepared VLPs contained highly pure F and M1 proteins (**Figure 2A**). Spherical self-assembling VLPs ~50-100 nm in diameter were observed under an electron microscope (**Figure 2B**). These data demonstrated that VLPs were successfully generated from rBV-infected Sf9 cells.

Previous studies and clinical observations in human sera demonstrated that RSV neutralizing antibodies are specific to the prefusion structure (22, 24, 26). To characterize the RSV Pre-F or Post-F form in the VLPs, the characteristic antigenic sites Ø and I based on the Pre-F and Post-F conformations were detected using the specific mAbs D25 and 131-2A, respectively. The data showed that mAb D25 bound effectively to VLPs containing Pre-F conformation with antigenic site Ø (**Figure 2C**, left). In contrast, mAb 131-2A bound strongly to VLPs containing the Post-F conformation with antigenic site I (**Figure 2C**, right). The results identified that the VLPs generated from rBV-Sf9 cells contained specifically antigenic sites Ø and I of the Pre-F and Post-F conformations, respectively.

### Antibody and cytokine responses in mice induced by Pre-F and Post-F VLPs

**Figure 3:**
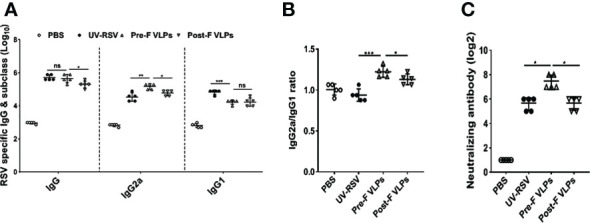
VLPs elicited humoral immune responses in mice. Groups of five BALB/c mice were inoculated with 10 μg of Pre-F VLPs, Post-F VLPs, 1×10^5^ PFU of purified UV-inactivated RSV or 100 μl PBS following i.m. inoculation. Mice in all groups received a booster twice in 2-week intervals with the same amount of inoculum. Sera were collected at 2 weeks after the final immunization to determine RSV-specific IgG, IgG2a, and IgG1 titres *via* ELISA **(A, B)** and neutralization antibody (NAb) titres *via* the neutralization assay **(C)**. Data are expressed as the GMT (geometric mean titre) of five mice. *P* values were calculated with one-way ANOVA or Student’s *t* test followed by the Tukey test. ***P < 0.001; **P < 0.01; *P < 0.05; ns, not significant.

RSV-specific IgG, IgG2a, and IgG1 concentrations in the sera of immunized mice were detected by ELISA. Compared with the Post-F VLPs, the Pre-F VLPs induced significantly increased RSV-specific IgG and IgG2a antibody levels (*P* < 0.05), but a similar IgG1 antibody level was observed (*P* > 0.05) (**Figure 3A**). Vaccination with the Pre-F VLPs and Post-F VLPs elicited Th1-dominant responses with median IgG2a/IgG1 ratios of 1.22 and 1.13, respectively; in contrast, immunization with UV-RSV resulted in a Th2-biased response with an IgG2a/IgG1 ratio of 0.94 (**Figure 3B**). Vaccination with Pre-F VLPs induced a higher ratio of IgG2a antibodies than vaccination with UV-RSV or Post-F VLPs. Sera of vaccinated mice exhibited stronger binding ability with the corresponding VLPs (**Supplementary Figure S2**). Analysis of RSV neutralizing antibody (NAb) in the sera of vaccinated mice showed that although both Pre-F VLPs and Post-F VLPs induced RSV NAb production, Pre-F VLPs elicited significantly increased RSV NAb levels compared with Post-F VLPs (*P* < 0.01) (**Figure 3C**).

**Figure 4:**
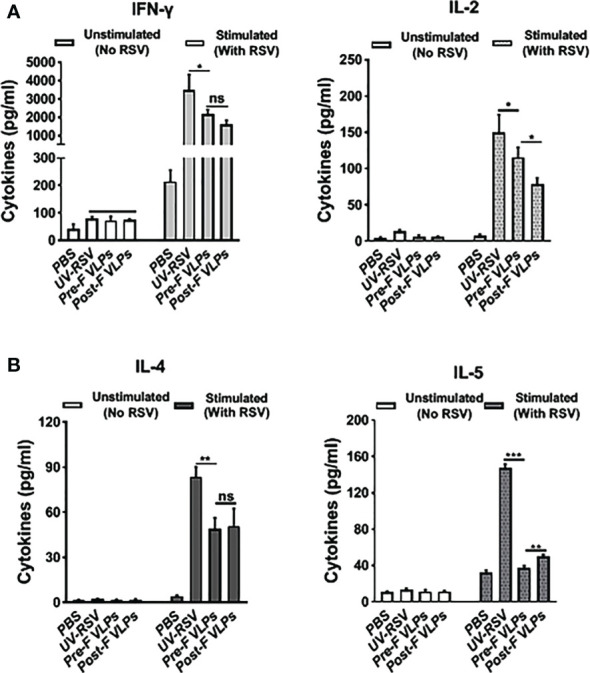
VLPs elicited cellular immune responses in mice. Spleen cells were isolated at 2 weeks after the last immunization and stimulated with 1×10^5^ PFU of purified UV-inactivated RSV. The supernatants were collected after 72 h of incubation, and Th1 cytokines (IFN-γ and IL-2) **(A)** and Th2 cytokines (IL-4 and IL-5) **(B)** concentrations were measured by ELISA. All data are presented as the mean values (± SD) from five mice in each group. *P* values were calculated with one-way ANOVA or Student’s *t test* followed by the Tukey test. ***P < 0.001; **P < 0.01; *P < 0.05; ns, not significant.

To investigate cellular immune responses, we examined Th1-type (IFN-γ and IL-2) and Th2-type (IL-4 and IL-5) cytokines in the splenocyte supernatants of vaccinated mice. In the PBS-treated group, both Th1-type (IFN-γ and IL-2) and Th2-type (IL-4 and IL-5) cytokines displayed very low concentrations. Compared with the VLP-vaccinated groups, significantly increased levels of Th1-type and Th2-type cytokines were induced by UV-RSV. However, in the VLP-vaccinated mice, the levels of the cytokines IFN-γ and IL-2 were only reduced approximately 1.4-fold, and the levels of the cytokines IL-4 and IL-5 were reduced ~2-fold and ~ 4-fold, respectively (**Figure 4**). Importantly, compared with Post-F VLPs, vaccination with Pre-F VLPs induced significantly increased Th1 type and decreased Th2-type cytokine production (**Figure 4**). Without RSV stimulation, both Th1-type and Th2-type cytokines were almost undetectable (**Figure 4**). Our data demonstrated that Pre-F VLPs elicited a mixed Th1/Th2 response with Th1-biased cellular immunity to RSV.

### CD4^+^ T-cell subsets and cytokine profiles in the lungs of vaccinated mice following RSV infection.

**Figure 5:**
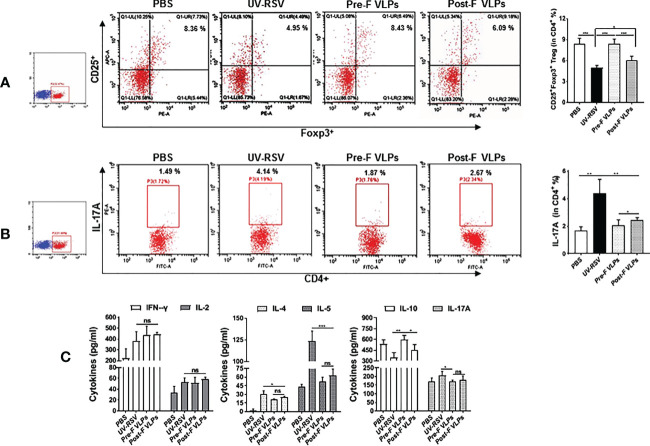
Cellular immune responses in the lungs of vaccinated mice induced by RSV challenge infection. Mice were immunized i.m. thrice and challenged i.n. with RSV 2 weeks after the final immunization. Lungs were harvested on Day 4 p.c. The percentage of CD25^+^ Foxp3^+^ Treg cells **(A)** or IL-17A **(B)** in CD4^+^ T cells from the lungs was measured by flow cytometry with specific antibody staining. **(C)** Th1 cytokine (IFN-γ & IL-2), Th2 cytokine (IL-4 & IL-5) and the cytokines IL-10 and IL-17A concentrations were measured by ELISA. Data are presented as mean values ± SDs for six mice in each group. Pairwise comparisons of values were performed using one-way ANOVA or Student’s *t* test followed by the Tukey test. ***P < 0.001; **P < 0.01; *P < 0.05; ns, not significant.

Distinct CD4^+^ T-cell subsets and Th2-type cytokines play crucial roles in RSV vaccine-enhanced immunopathology (13, 14, 47, 51). We further investigated CD4^+^CD25^+^Foxp3^+^ Treg and IL-17A-producing CD4^+^ T-cell subsets and the representative cytokines in the lungs of vaccinated mice following RSV challenge. The data showed that the percentage of CD4^+^CD25^+^Foxp3^+^ Treg cells in VLP-vaccinated mice was significantly increased compared to that in UV-RSV-immunized mice (*P* <0.001) (**Figure 5A**). In contrast, significantly decreased IL-17A^+^-producing CD4^+^ T cells were observed in VLP-vaccinated mice compared to UV-RSV-immunized mice (*P* < 0.01) (**Figure 5B**). In particular, a significantly increased percentage of CD4^+^CD25^+^Foxp3^+^ Treg cells (*P* < 0.01) (**Figure 5A**) and a significantly decreased amount of IL-17A^+^-producing CD4^+^ T cells (*P* < 0.05) (**Figure 5B**) were observed in mice vaccinated with Pre-F VLPs compared to mice vaccinated with Post-F VLPs. Similar concentrations of the pulmonary cytokines IFN-γ, IL-2, IL-5 and IL-17A were observed in mice vaccinated with Pre-F compared with Post-F VLPs **Figure 5C**. Importantly, significantly decreased concentrations of the Th2-type cytokines IL-4 (*P* < 0.05) and IL-5 (*P* < 0.001) and significantly increased production of the Treg cell-related cytokine IL-10 (*P* < 0.01) were observed in VLP-vaccinated mice compared to UV-RSV-immunized mice **Figure 5C**. Interestingly, compared to Post-F VLPs, vaccination of Pre-F VLPs elicited significantly decreased IL-4 (*P* < 0.01) and increased IL-10 secretion (*P* < 0.05) (**Figure 5C**). Our results indicated that Pre-F VLPs elicited balanced Treg/Th17 responses in vaccinated mice upon subsequent RSV infection.

### Pulmonary viral load and pathology in mice vaccinated with VLPs following RSV infection

**Figure 6:**
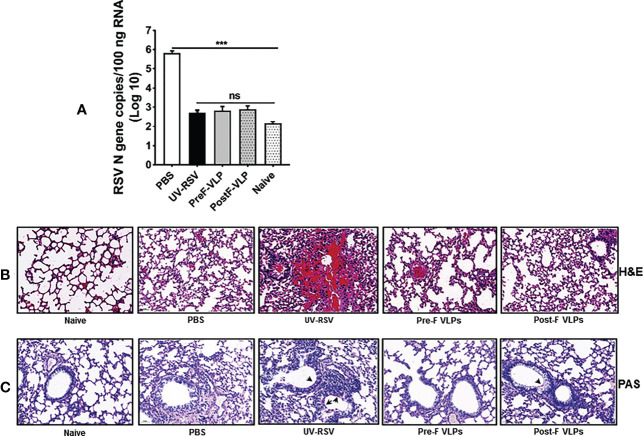
RSV load and histopathological analysis of lung tissues from vaccinated mice upon RSV challenge. Mice were immunized i.m. thrice and challenged i.n. with RSV 2 weeks after the final immunization. Lungs were harvested on Day 4 p.c. **(A)** RSV copy numbers in lung tissues were measured by qRT−PCR. Data are presented as mean values ± SDs for six mice in each group. Pairwise comparisons of values were performed using one-way ANOVA followed by the Tukey test. ***P < 0.001; **P < 0.01; *P < 0.05; ns, not significant. **(B, C)** Representative histopathological section of lung from immunized mice at Day 4 after RSV challenge by haematoxylin-eosin (H&E) **(B)** and periodic acid-Schiff (PAS) staining **(C)** for each experimental group.

To investigate the immune protection induced by the VLPs, the RSV load in the lungs of vaccinated mice was quantified on Day 4 p.c. by qRT−PCR. As a control, the high RSV N gene copy numbers (~ 10^6^ copies/100 ng total RNA) were detected in the lungs of PBS-treated mice, whereas very low RSV N gene copy numbers (~ 10^3^ copies) (approximate background value of test) were observed in the lungs of mice vaccinated with VLPs (**Figure 6A**), demonstrating that vaccination with Pre-F VLPs or Post-F VLPs effectively inhibited RSV replication in the lungs of mice.

We further investigated pathological injury in the lungs of vaccinated mice after RSV infection. The data showed that mice immunized with UV-RSV exhibited severe lung pathology, including extensive lymphocyte infiltration around the blood vessels and alveolar hemorrhage. In contrast, mice vaccinated with VLPs displayed signs of mild inflammation in the lungs. Importantly, mice vaccinated with Pre-F VLPs presented similar histological features to naive mice in the lungs (**Figure 6B**). After RSV infection, the average inflammation severity scores of vaccinated mice were in the following order: UV-RSV > PBS > Post-F VLPs > Pre-F VLPs (**Table 2**). Data from PAS staining showed that overt inflammation and mucus hypersecretion (black arrows) were observed in the lungs of UV-RSV-immunized mice and mild mucus accumulation was observed in the lungs of Post-F VLP-immunized mice (**Figure 6C**). However, no mucus secretion was observed in the lungs of mice vaccinated with Pre-F VLPs, which was similar to the characteristics of naive mice. These results revealed that vaccination with Pre-F VLPs induced effective protection against RSV infection without enhanced pulmonary immunopathology in mice.

**Table 2:** Histopathological scores of lungs from immunized mice on Day 4 following RSV challenge.

	Histopathological score^a^			
Inoculum	Alveolartissue^b^	Peribronchial aggregation^c^	Perivascular aggregation^d^	Mucus^e^
Naive	0	0	0	1
PBS	2 ± 0.16	2.2 ± 0.16	2.27 ± 0.19	1.33 ± 0.09
UV-RSV	3.67 ± 0.09	3.67 ± 0.09	3.53 ± 0.09	2.6 ± 0.16
Pre-F VLPs	0.67 ± 0.09	0.73 ± 0.09	0.8 ± 0.16	1.13 ± 0.09
Post-F VLPs	1.33 ± 0.09	1.53 ± 0.25	1.53 ± 0.09	1.4 ± 0.16

a The lung inflammation severity scores were defined on a scale from 0 to 4 according to the H&E-stained sections as follows: 0-inflammation was not present, 1- minimal inflammation, 2-mild inflammation, 3-moderate inflammation, and 4-marked inflammation. Scores of mucus production scale according to the PAS-stained sections as follows: 1-no mucus detectable, 2-rare mucus, 3-moderate mucus accumulation, and 4-severe mucus accumulation. Data represent the mean values ± SDs (n = 3).

b Alveolar tissue: Pre-F VLPs vs. Post-F VLPs (p < 0.01).

c Peribronchial aggregation: Pre-F VLPs vs. Post-F VLPs (p < 0.05).

d Perivascular aggregation: Pre-F VLPs vs. Post-F VLPs (p < 0.05).

e Mucus: Pre-F VLPs vs. Post-F VLPs (p > 0.05).

## Discussion

A licensed vaccine for RSV is not currently available despite the fact that RSV is the major cause of lower respiratory tract infections in children. Vaccine-enhanced immunopathology has significantly hampered the development of an RSV vaccine. Previous studies have shown that poorly neutralizing antibodies, a Th2-biased immune response and distinct CD4^+^ T-cell subsets correlate with VED upon RSV infection (12, 13, 47, 52) and that high neutralizing antibody levels correlate with the prevention of disease severity and a lower risk of infection (6, 53, 54). Therefore, induction of a high-affinity neutralizing antibody and a balanced immune response should be preferentially considered for the design of a safe and effective RSV vaccine (18, 55).

Experimental studies and clinical observations in human sera demonstrated that RSV neutralizing antibodies are specific to the prefusion structure (22, 24, 26). Structure-based design of vaccines showed that a highly stable prefusion F conformation would be a promising subunit vaccine candidate against RSV (27, 29). Following immunization of mice, VLPs containing the stabilized Pre-F configuration from Newcastle disease virus (NDV) induced significantly higher neutralizing antibody titres than the Post-F VLPs or wild-type F VLPs after a single immunization (49). As a well-known tool for the production of subunit vaccines, the rBV-Sf9 insect cell expression system has been widely employed (56–58). VLPs containing the RSV F protein generated by rBV-Sf9 cells confer effective protection against RSV infection (37, 38). In the present study, we successfully produced influenza M1-based VLPs containing RSV Pre-F or Post-F configuration using the rBV-Sf9 cell expression system. We further characterized the assembly and immunological properties of these VLPs. Our results confirmed that the Pre-F and Post-F VLPs generated from rBV-Sf9 cells displayed specific antigenic sites Ø and I, respectively. The antigenic site Ø was the crucial target site recognized by RSV-neutralizing antibodies, and the postfusion form of the F protein led to the lack of specific epitopes Ø (22, 26, 27). Antigenic site I was more pronounced on Post-F, and site I-directed antibodies are typically nonneutralizing (59). Our results demonstrated that Pre-F VLPs elicited significantly increased RSV-specific neutralizing antibody titres compared to PostF VLPs or UV-RSV. The high level of Pre-F specific antibodies in human sera play an important role in alleviating antibody-dependent disease enhancement (60–62).

In our study, immunization with both Pre-F and Post-F VLPs predominantly induced IgG2a isotype antibodies and Th1-associated cytokines (IFN-γ and IL-2) and significantly decreased Th2-biased cytokine responses (IL-4 and IL-5) compared with UV-RSV. For cytokine secretion in the lungs of mice vaccinated with VLPs, significantly decreased IL-4, IL-5, and IL-17A and increased IL-10 cytokines were observed compared to UV-RSV. Importantly, compared with Post-F VLPs, Pre-F VLPs elicited a significantly increased IgG2a/IgG1 ratio and high RSV neutralizing antibody levels and significantly decreased IL-5 secretion. However, both Pre-F VLPs and Post-F VLPs induced similar IFN-γ and IL-4 cytokine levels (**Figure 4**). We further tested the Treg and Th17 subsets in the lungs of vaccinated mice. Compared to UV-RSV, vaccination with RSV F VLPs upregulated the percentage of Treg (CD4^+^CD25^+^FoxP3^+^) cells and downregulated the percentage of Th17 (CD4^+^IL-17A^+^) cells. Significantly increased Treg cells and decreased Th17 cells were observed in the lungs of mice vaccinated with Pre-F VLPs compared with Post-F VLPs. During RSV infection, Treg cells functionally regulate the immunological environment to avoid excessive inflammatory T-cell responses and aid in limiting inefficient Th2-type immune responses (55). Therefore, Treg cells play a pivotal role in alleviating vaccine-enhanced immunopathology in RSV infection (55, 63). In contrast, Th17 cells are functionally considered to exacerbate inflammatory diseases, including chronic pulmonary obstruction, cystic fibrosis, and asthma (64), and are involved in increasing mucus secretion and reducing viral clearance (65). A current study showed that vaccination with commercial PreF protein formulated with a Th1/Th2-balanced adjuvant induced suppression of RSV replication and inhibited airway eosinophilia and mucus accumulation in mice (66). Additionally, poor avidity and affinity maturation caused nonprotective antibody development and Th2-associated immunopathology (52). As expected, our results demonstrated that a high neutralizing antibody level and a Th1/Th2-balanced immune response were induced by Pre-F VLPs, resulting in alleviation of pulmonary pathology and airway mucus secretion in vaccinated mice.

VLPs containing RSV F and/or G proteins have been intensively investigated using different vaccine platforms (18, 37, 39, 40, 44, 67). For NDV-RSV VLPs, the potential contamination of mammalian DNA and other deleterious factors from sera and/or cells require removal from VLPs (49, 68). The rBV-produced VLPs from serum-free insect cell cultures are beneficial to VLP vaccine technology. RSV VLP production utilizing the rBV-insect cell expression system is FDA approved for human use, and the high levels of VLPs generated from suspension cultures of insect cells will facilitate large-scale vaccine production (57, 58, 69). Therefore, the work presented in this study provides a novel, promising strategy for the development of RSV vaccine candidates.

## Data availability statement

The original contributions presented in the study are included in the article/supplementary materials, further inquiries can be directed to the corresponding author/s.

## Ethics statement

The animal study was reviewed and approved by the Institutional Animal Care and Use Committee of Wuhan University.

## Author contributions

ZP, JL, and LL designed and conceived the study. JL, HQ, WL, and RL conducted the experiments. ZP, JL, and LL analyzed the data and wrote the manuscript. All authors contributed to the article and approved the submitted version.
