# Supplementary material for: Virus-like particles containing a prefusion-stabilized F protein induce a balanced immune response and confer protection against respiratory syncytial virus infection in mice
Source: Front Immunol. 2022 Dec 12;13:1054005. doi: 10.3389/fimmu.2022.1054005 (PMC9792133; doi:10.3389/fimmu.2022.1054005)
Supplement: Supplementary file 1 [file DataSheet_1.pdf]

Supplementary Materials

1. The gating procedure for flow cytometry analysis of Treg and Th-17 subclass CD4$^+$ T cells.

For flow cytometric analysis, the gating strategy of stained cells was performed as the recommendation's protocol. For Treg analysis, the stained cells were first gated based on size and granularity followed by singlet cells based on FSC-A vs. FSC-H and FSC-W vs. SSC-H. Then, the CD4 (in FITC) positive T cells were gated. Finally, a quad gate for screening Foxp3$^+$ (in PE) and CD25$^+$ (in APC) cells. Similarly, for Th17 analysis, the stained cells first gated based on size and granularity followed by singlet cells based on FSC-A vs. FSC-H and FSC-W vs. SSC-H, and subsequently the CD4$^+$ T (in FITC) cells were gated. Finally, these gated cells were analysed for IL-17A$^+$ (in PE) cells.

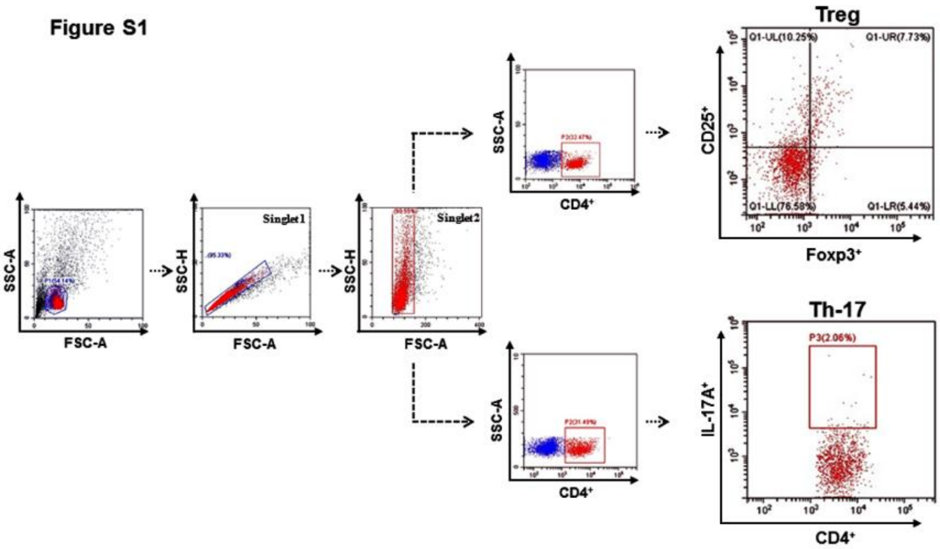

Figure S1. The gating procedure for flow cytometry analysis of T CD4$^+$ T cells

2. The expressed proteins reacted specifically with sera of vaccinated mice. To evaluate the reactivity of the expressed proteins, we examined the VLP binding ability with the serum antibodies of experimental mice using ELISA based on Pre-F or Post-F VLPs as coating antigen.

Expectedly, the purified VLPs exhibited specifically high binding ability with the serum antibody from corresponding mice (**Figure S2**).

Figure S2

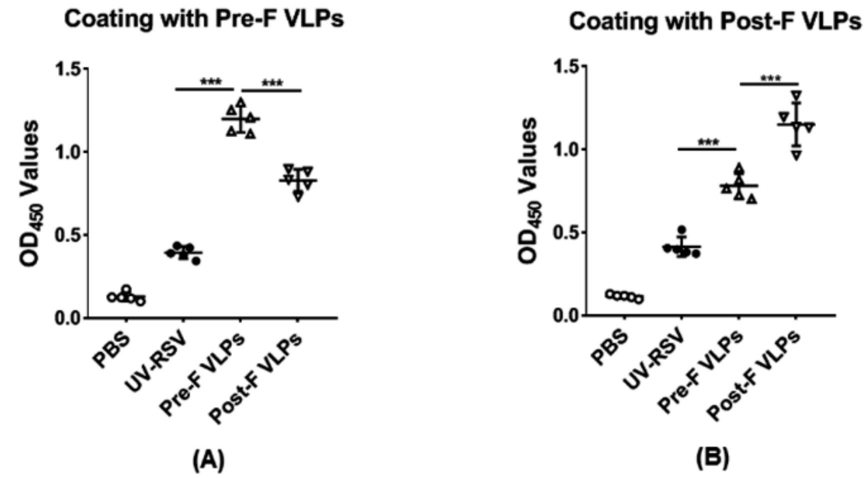

Figure S2. The VLP binding ability with sera of vaccinated mice. BALB/c Mice were immunized as described in Materials and Methods. Sera of immunized mice were collected at 2 weeks after the final immunization, respectively. The reactivity of the expressed proteins with sera of immunized mice was evaluated by ELISA. (A) Purified Pre-F VLPs were used as coating antigen; (B) Purified Post-F VLPs were used as coating antigen. ***, $P < 0.001$.
